# Unusual Presentation of Prostate Cancer: A Case Report

**DOI:** 10.7759/cureus.49779

**Published:** 2023-12-01

**Authors:** Mariana Pinto, José Diez Carvalho, Gonçalo Magalhães, Sílvia Gomes, Filipa Figueiredo

**Affiliations:** 1 Family and Community Medicine, Unidade de Saúde Familiar (USF) Infante D. Henrique (Family Health Unit Infante D. Henrique), Viseu, PRT; 2 Family and Community Medicine, Unidade de Saúde Familiar (USF) Coração da Beira (Family Health Unit Coração da Beira), Canas de Senhorim, PRT

**Keywords:** primary healthcare services, meningioma, adenocarcinoma, brain metastases, prostate cancer

## Abstract

Prostate cancer is the second-most common malignancy in males. Despite more frequently metastasizing to the bone, regional lymph nodes, and liver, the brain can also be affected. These metastases can simulate meningiomas, making the diagnosis more difficult. Here, we report the case of a 62-year-old male with a sudden onset of confusion and dysarthria with spontaneous resolution but amnesia for the event. On a neurological exam, the patient had left exophthalmos and palpebral ptosis. He was referred to the emergency room, where he underwent a cranioencephalic CT, which revealed a left anterior temporal lesion with adjacent edema suggestive of meningioma, later confirmed by an MRI. Due to the worsening of the symptoms and an increase in the size of the lesion, total resection was proposed. The anatomopathological study revealed a poorly differentiated carcinoma. To study the primary tumor, a CT of the thorax, abdomen, and pelvis; a spine MRI; and a complementary study with prostate-specific antigen were requested. These studies revealed a prostate adenocarcinoma with brain and bone metastases. After the diagnosis, the patient underwent hormone therapy, chemotherapy, and palliative radiotherapy.

## Introduction

Prostate cancer is the second most common malignant neoplasm in men, accounting for 14.1% of all neoplasms diagnosed in 2022, only surpassed by lung cancer (14.3%). The mortality rate (around 7%) is lower than that of lung, liver, and stomach cancer [1]. When it metastasizes, it often affects the bone, nearby lymph nodes, and liver [2]. In less than 1% of cases of metastasized prostate cancer, there is brain spread [2,3]. This can cause neurological symptoms and can be mistaken for primary brain tumors such as meningiomas [2], which are the most common benign intracranial tumors. This can delay diagnosis and prevent a timely therapeutic approach. Therefore, this case report aims to alert health professionals to the less common presentations of prostate cancer, focusing on the importance of recognizing warning signs and prompt action by the attending physician.

## Case presentation

The patient is a Caucasian, 62-year-old male, and was the head of the finance department of a company. He belonged to an extended family (stage VII of the Duvall family lifecycle) and was of medium-high socioeconomic class, according to the adapted Graffar Scale. His personal medical history included dyslipidemia, overweight (BMI 30.3 kg/m2), and atrial flutter, for which he underwent ablation in 2019. His prostate cancer screening didn't reveal any alteration, with stable prostate-specific antigen (PSA) values of around 2 ng/mL since 2013. Surgical history included the placement of a left hip prosthesis in 2009, a tonsillectomy more than 30 years ago, and the removal of a vocal cord polyp. He also had a drinking habit (10 drinks a day) and a history of smoking, with a smoking load of 75 pack years, and has been abstinent since 2019. His daily medication included metoprolol, rosuvastatin, omeprazole, and furosemide. His family history included a daughter with Hodgkin's lymphoma diagnosed at the age of 12 and the death of his mother at the age of 74 from nasopharyngeal cancer.

In March 2022, he consulted an ophthalmologist for left eyelid swelling and was treated with topical corticosteroids. In April 2022, he consulted his family doctor due to a sudden onset of confusion and dysarthria earlier that day, lasting one minute and with spontaneous resolution, but with amnesia about what had happened, according to his daughter's statement. Upon objective examination, only the presence of slight exophthalmos and left eyelid ptosis stood out, with no other noticeable deficits found during the neurological examination.

**Figure 1:**
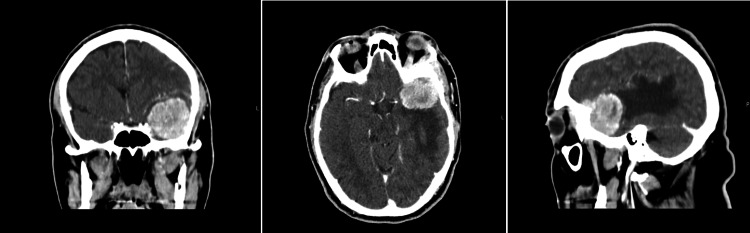
Coronal, cross-sectional, and sagittal views of the head CT scan sections showing a space-occupying lesion

Given this situation, the patient was referred to the emergency room, where he underwent a cranial-encephalic CT scan that revealed a left anterior temporal lesion with adjacent edema, compatible with meningioma, which was later confirmed on an MRI. The diagnostic hypothesis was episodic motor dysphasia in the context of focal crisis and left temporal meningioma. He was treated with antiepileptic drugs and corticosteroids. In June 2022, due to the worsening of his visual and neurological deficits, he underwent a new CT scan, which revealed an increase in the size and edema of the lesion (Figure 1).

Due to clinical and imaging deterioration, the patient underwent a left fronto-temporo-orbital craniotomy the following week. In July 2022, he underwent a control CT scan, which showed practically complete regression of the cerebral edema. Subsequently, the anatomopathological study revealed that it was a poorly differentiated carcinoma. The immunohistochemical profile revealed cancer cells with large expression of CK8/18 and AE1/AE3, focal expression of epithelial membrane antigen (EMA), and no expression of CK7, TTF-1, SSTR-2a, P63, CK5/6, S100 protein, or MELAN-A. The Ki-67 proliferation index was 30%.

After establishing this diagnosis, a search for primary pathology was carried out with a CT thorax, abdomen, and pelvis (TAP) scan. The CT TAP revealed, as relevant alterations, signs of left frontotemporal craniotomy with a persisting collection of heterogeneous content, but with gas bubbles in the left retro-orbital region with peripheral contrast uptake that is probably related to inflammatory alterations; multiple punctate pulmonary micronodules with some of high-density that may only be residual, the rest being non-specific; the presence of two tiny millimetric hypodensities in the liver up to 3 mm: one in the caudate lobe and the other on the periphery of segment IV, which is too small for proper characterization but could correspond to small cysts; prostate and seminal vesicles were totally inconclusive, requiring further investigation; and, finally, there were signs of diffuse bone metastasization with multiple bone metastases of a blastic predominance, although with some lytic images, particularly in the left iliac bone adjacent to the prosthesis, which could also be metastatic.

**Figure 2:**
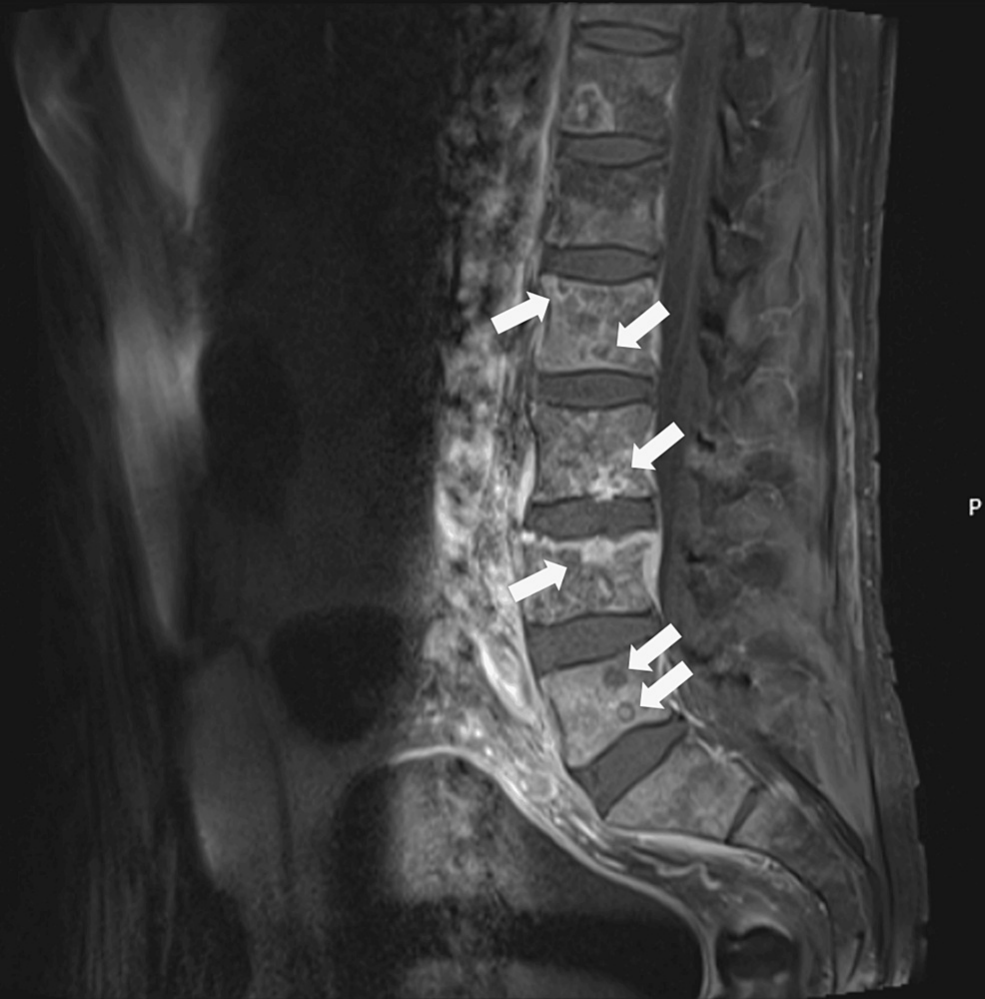
MRI section of the lumbosacral spine with foci of metastization (white arrows)

It is important to note that, at this stage, the patient had gait impairment due to pain that started two weeks before. The analytical study revealed increasing PSA values, rising from 3.17 ng/mL at the end of January (around two months before he started complaining of left eyelid edema) to 91.4 ng/mL at the beginning of August. A transrectal prostate biopsy confirmed the diagnosis of stage IV prostate adenocarcinoma (Gleason score of 8) with brain and bone metastization. Figure 2 shows foci of heterogeneous T1 hyposignal in the vertebral pieces, reflecting extensive bone metastization. Also visible in L4 was the thickening of the paravertebral soft tissues associated with bone metastasis and depression of the upper platform of this vertebra, suggesting the coexistence of a recent pathological fracture.

**Figure 3:**
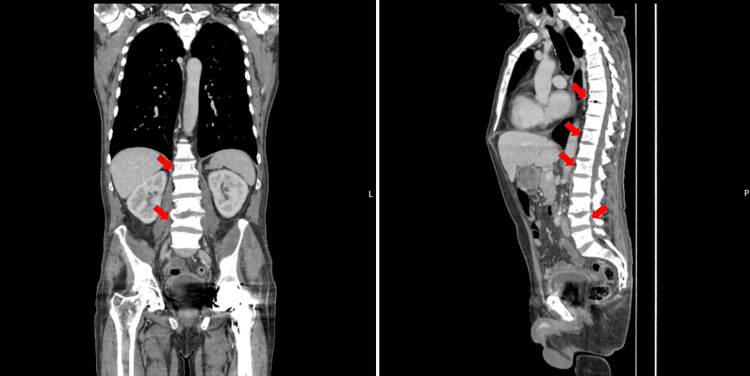
CT TAP showing foci of metastization and flattening of L4 vertebra TAP: Thorax, abdomen, and pelvis

It was possible to control the lumbar pain with opioids and corticosteroids. The exophthalmos had also regressed almost completely. Following a therapeutic decision by the attending medical team, the patient underwent cycles of chemotherapy with docetaxel and adjuvant radiotherapy. In November, due to rising PSA levels, he underwent a new CT TAP scan and bone scintigraphy for re-staging. Both tests revealed signs of diffuse bone metastization, with an increase in the number of lesions and the growth of pre-existing ones. Figure 3 shows some of these lesions and a new flattening of the L4 vertebral body in relation to a probable pathological fracture.

The patient is currently being followed up by oncology, urology, and neuro-ophthalmology and is maintaining treatment for the prostate adenocarcinoma as well as pain control through opioids and corticosteroids, along with regular imaging and analytical follow-up. It should be noted that, in a recent consultation with his GP, the patient downplayed his prognosis, calling on the doctor to ensure the well-being of his daughter, who had been diagnosed with Hodgkin's lymphoma at the age of 12, trying to focus the main concern on her. This highlights the role of the GP as the 'patient's and family's advocate,' who must respect each patient's decision and act to ensure their well-being, whether this involves them or, as in this case, third parties.

## Discussion

Prostate cancer is the second most common malignant neoplasm in men, after lung cancer. However, in men of African ethnicity, it is the main malignant neoplasm, and in the United States of America, it accounts for 20% of the total incidence of male cancer, higher than 13% of lung cancer [1]. The incidence rate is strongly related to age, as it is the main risk factor for prostate cancer along with ethnicity and family history, with a significant rise in age-specific rates from the age of 50 [4].

According to 2022 data from the European Cancer Information System (ECIS), prostate cancer had an average incidence rate of 154.1 new cases per 100,000 inhabitants in the 27 countries of the European Union (EU), with a mortality rate of 38.6 per 100,000. In Portugal, the incidence rate is lower than the EU average, but the mortality rate is higher (39.8 deaths per 100,000 inhabitants) [5]. Screening through PSA assessment remains controversial. Although there are studies that estimate that elevations in PSA can precede clinical manifestations of prostate cancer by five to 10 years [6,7], it is known that it can be elevated in the absence of prostate cancer in men with benign pathology (such as benign prostatic hyperplasia) or acute pathology (such as prostatitis). However, the rate of false negatives should also be taken into account, as reports show that it could miss 15% of prostate cancers [8]. In this case, the patient had a PSA value of 3.17 ng/mL in January 2022, about two months before the first complaint of eyelid edema and three months before the first imaging evaluation that revealed the brain tumor, later assumed to be a metastasis of prostate adenocarcinoma.

At a clinical level, most diagnoses are made in men with lower urinary tract symptoms (LUTS) such as nocturia, pollakiuria, or difficulty initiating urination. Although there is some evidence of an increased risk of localized prostate cancer in men with LUTS, there has been no association between LUTS and advanced prostate cancer. This may be because these men are more likely to have an analytical study that can identify this neoplasm at an early stage, which may or may not affect their long-term survival [9].

The organs most affected by prostate cancer metastases are the bone (84%), lymph nodes (10.6%), liver (10.2%), and chest (9.1%) [10]. It is estimated that brain metastases occur in less than 1% of prostate cancer cases, with a study involving 4341 Australian men with this cancer revealing intracranial metastases in only eight, resulting in an incidence of 0.18% [3]. On the other hand, brain metastases are mainly related to lung cancer (24.1%), followed by skin cancer (7.3%), cancer of unknown primary origin (3%), and only then prostate cancer (2.7%) [11]. In this patient's case, and given the absence of urinary symptoms that could have led to suspicion of a prostatic lesion, the lesion found on the CT scan was initially assumed to be a meningioma, as this is the most common primary intracranial tumor. Distinguishing between a primary brain lesion and a prostate cancer metastasis is difficult, especially when a solitary lesion is found. Only the anatomopathological study allowed the diagnosis to be made in this case, although the literature reveals cases in which the biopsied sample was positive for meningioma despite being a metastasis [2]. The subsequent imaging and analytical study proved to be essential for staging and defining the treatment to be followed; in this case, bone metastasis was identified, a more common occurrence in this type of malignant neoplasm and which, in the post-diagnostic phase, caused and continues to cause the patient to complain of low back pain, which is only controlled with opioids and corticosteroids.

## Conclusions

The rarity of this manifestation in a patient and the non-specific symptomatology for this pathology can delay the diagnostic process and, subsequently, a timely therapeutic decision aimed at prolonging the patient's survival. It is therefore essential for the doctor to be attentive to each patient's signs and symptoms, combined with an extensive and careful anamnesis; to consider carrying out analytical tests that can help with this surveillance; and to act even if less likely diagnostic hypotheses are taken into account. Therefore, the publication of this case aims to raise awareness of possible rare manifestations of a common malignant neoplasm so that it can be diagnosed as early as possible, enabling the patient to receive appropriate treatment and follow-up, improving the prognosis.
